# RM2 and DB15 analogues bearing [^177^Lu]Lu-DOTAGA via different linkers, as radiotherapeutics: a head-to-head comparative study

**DOI:** 10.1186/s41181-025-00374-3

**Published:** 2025-07-31

**Authors:** Panagiotis Kanellopoulos, Athanasios Bitzios, Ivan Zelepukin, Ekaterina Bezverkhniaia, Theodosia Maina, Berthold A. Nock, Vladimir Tolmachev, Anna Orlova

**Affiliations:** 1 Department of Medicinal Chemistry, Uppsala University, 751 23 Uppsala, Sweden; 2 Molecular Radiopharmacy, INRaSTES, NCSR “Demokritos”, 15341 Athens, Greece; 3 Department of Immunology, Genetics and Pathology, Uppsala University, 751 83 Uppsala, Sweden; 4 Science for Life Laboratory, Uppsala University, 752 37 Uppsala, Sweden

**Keywords:** GRPR, Bombesin, Lu-177, Radiotherapy, PRRT, Pharmacokinetics

## Abstract

**Background:**

Bombesin analogues are gaining popularity as GRPR-targeting theranostic agents aiming to provide molecular tools for a patient-tailored management. We previously reported on two series of DOTAGA-bearing GRPR-antagonists, based on either [NMe-Gly^11^]RM26 (DOTAGA-X-DPhe-Gln-Trp-Ala-Val-NMe-Gly-His-Sta-Leu-NH_2_) or on DB15 (DOTAGA-X-SAR; SAR: DPhe-Gln-Trp-Ala-Val-NMe-Gly-His-Leu-NHEt) motifs, which were preclinically screened after labelling with In-111. In the current study, we aimed to evaluate in vitro and in vivo the four best-performing agents, AU-RM26-M2 (X: PEG2-Pip; Pip: 4-amino-1-carboxymethyl-piperidine), AU-RM26-M4 (X: Arg-Arg-Pip), AU-SAR-M1 (X: AMA-DIG; AMA: p-amino methylaniline, DIG: diglycolate) and AU-SAR-M2 (Arg-AMA-DIG), this time labelled with the therapeutic radionuclide Lu-177.

**Results:**

All four [^177^Lu]Lu-peptide radioligands displayed highly GRPR-mediated cellular uptake, showing the typical profile of radioantagonists, with the bulk of cell-associated radioactivity being membrane-bound. The analogues demonstrated good in vivo stability, which was however further improved by in situ stabilization induced by pretreatment of animals with Entresto as the source of the potent neprilysin (NEP)-inhibitor sacubitrilat. The biodistribution profile of the four radiopeptides was determined in prostate cancer PC-3 xenograft-bearing mice at 4 h and 23 h pi, after Entresto pre-treatment. All peptide radioligands had a rapid clearance from the background tissues, with the highest activity uptake found in the implanted tumours, the kidneys and to a lesser extent the GRPR-rich pancreas. The activity in the pancreas and, on a smaller scale, in the kidneys was washed out by 23 h pi, while being highly retained in the tumours. Among the tested analogues, [^177^Lu]Lu-AU-SAR-M1 displayed the overall most favourable properties, combining the lowest retention in the kidneys with high and prolonged activity accumulation in the tumours. As a result, [^177^Lu]Lu-AU-SAR-M1 provided the best area under the curve (AUC) ratio between tumour and kidneys (5.4), in comparison with [^177^Lu]Lu-AU-SAR-M2 (3.8), [^177^Lu]Lu-AU-RM26-M4 (3.4), and [^177^Lu]Lu-AU-RM26-M2 (1.1).

**Conclusions:**

In conclusion, these results qualify [^177^Lu]Lu-AU-SAR-M1 as the candidate of choice for further evaluation in a dedicated preclinical radiotherapy study.

**Supplementary Information:**

The online version contains supplementary material available at 10.1186/s41181-025-00374-3.

## Background

The gastrin releasing peptide receptor (GRPR) still keeps attracting the spotlight as a valid biomolecular target for the development of novel radiopharmaceuticals for personalized management of a wide range of cancer patients. These include GRPR-expressing gastrinoma, breast and prostate carcinoma, or small cell lung cancer patients (Mattei et al. 2014; Morgat et al. 2017; Nock et al. 2005; Reubi et al. 2002a, b, 2004; Reubi 2007). Hence, GRPR-directed radioligands may be developed into potent radiotheranostic molecular tools in a multitude of applications in nuclear medicine, according to precision medicine principles. Indeed, the GRPR has been extensively investigated as a target since the 1990s (Mansi et al. 2021), and many analogues have made it into clinical trials. However, there is always space for improvements with respect to in vivo stability, pharmacokinetics or biosafety (Bodei et al. 2007; Cescato et al. 2008; Linder et al. 2009; Lymperis et al. 2018).

Many attempts have been made to remedy the abovementioned drawbacks. Thus, the issue of acute side effects elicited after systemic administration of GRPR-agonists was tackled by shifting from GRPR-agonists to antagonists (Baum et al. 2007; Bodei et al. 2007; Cescato et al. 2008; Nock et al. 2023). This approach quenched any further concerns rising from mitogenic effects of GRPR agonists as well (Farias et al. 2008; Hohla et al. 2007; Moody et al. 2003; Qiao et al. 2008; Rozengurt et al. 1990; Sun et al. 2023). As an additional benefit, GRPR-radioantagonists were often shown to have superior pharmacokinetics in comparison with their agonist counterparts (Cescato et al. 2008; Lymperis et al. 2019; Nock et al. 2023). Furthermore, various structural interventions with unnatural amino acids introduced in the peptide chain and/or different linkers connecting the receptor-targeting moiety with the radiometal-chelate were employed in an attempt to increase in vivo stability and improve pharmacokinetics (Baun et al. 2024; D’Onofrio et al. 2023; Dalm et al. 2024; Zou et al. 2024). As a result, several of GRPR-antagonists were introduced after proper modification on the C-terminal dipeptide fragment (Leu-Met-CONH_2_) of amphibian bombesin (BBN) and in particular the BBN(6–14) nonapeptide (de Castiglione and Gozzini 1996; Nock et al. 2017). The majority of these analogues were designed for theranostic use, utilizing a variety of radionuclides (Baun et al. 2024; D’Onofrio et al. 2023; Dalm et al. 2024; Zou et al. 2024). Peptidomimetic analogues, like RM2 (Damiana et al. 2023; Kurth et al. 2020), LF1 (Hofstetter et al. 2020), LE1 (Li et al. 2024; Wong et al. 2022), DOTAM-GRPR1 (Saidi et al. 2024), AMTG (Kurth and Al 2024) and NeoBomb1 (Dalm et al. 2017; Damiana et al. 2023; Montemagno et al. 2021; Nock et al. 2017; Ruigrok et al. 2022; Sabahi et al. 2025; Verhoeven et al. 2024), labelled with particle emitting radionuclides, such as Lu-177, Cu-64/67 and Pb-212, were tested for therapeutic purposes. In addition, some of these analogues have also been evaluated as diagnostic tools, labelled with radionuclides, such as Ga-68, Cu-61/64, for PET imaging (e.g. LE1 (Wong et al. 2022), RM2 (Beheshti et al. 2022; Duan et al. 2024; Ghezzo et al. 2024; Schollhammer et al. 2023), NOTA-PEG3-RM26 (Wang et al. a, b), AMTG (Urena Poch and Al. 2024), NOTA-PEG2-RM26 (Bjäreback et al. 2025; Csismazia et al. 2024), SB3 (Bakker et al. 2021) and NeoBomb1 (Gruber et al. 2022, 2020; Nock et al. 2017), as a means of diagnosis and follow-up of the therapeutic outcome. Notwithstanding, the search for Tc-99 m-based GRPR-directed radioligands is still prominent, with analogues like [^99m^Tc]Tc-BD8 (ongoing clinical study, NCT05940298), [^99m^Tc]Tc-DB15 (Nock et al. 2021), and [^99m^Tc]Tc-maS3-PEG2-RM26 (Chernov et al. 2023) being tested for SPECT imaging, which remains the most widely used imaging modality of nuclear medicine worldwide.

As part of our efforts to deliver stable and clinically effective GRPR-specific theranostic radioligands, we recently investigated two series of analogues, based on: a) the NMe-Gly ^11^-modified JMV594/RM26 (DPhe-Gln-Trp-Ala-Val-Sar-His-Sta-Leu-NH_2_; NMe-Gly: sarcosine) and b) SAR (DPhe-Gln-Trp-Ala-Val-NMe-Gly-His-Leu-NHEt). All members bear the DOTAGA-chelator (DOTAGA: 1,4,7,10-tetraazacyclododecane-1-glutaric acid-4,7,10-triacetic acid) via different linkers and were labelled with In-111 (Abouzayed et al. 2023; Kanellopoulos et al. 2024; Obeid et al. 2024). The incorporation of positively charged linkers was shown to increase the receptor affinity, however often at the cost of in vivo stability.

**Fig. 1:**
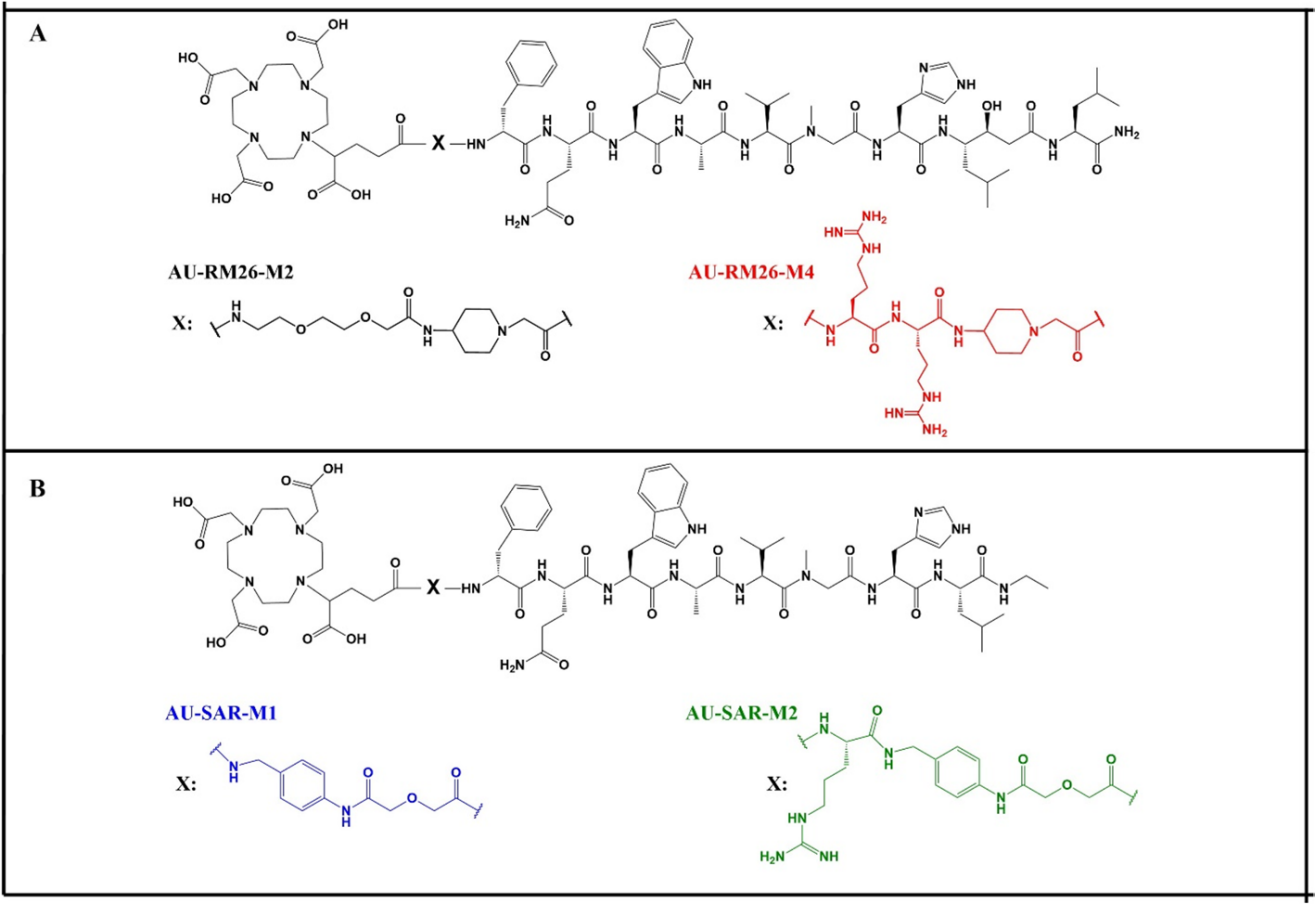
Chemical structures of **a** AU-RM26-M2 (X: PEG2-Pip), AU-RM26-M4 (X: Arg-Arg-Pip); **b** AU-SAR-M1 (X: AMA-DIG) and AU-SAR-M2 (X: Arg-AMA-DIG)

Among these two compound libraries, comprised of seven members in total, four analogues showed the best biological performance: i. AU-RM26-M2 (DOTAGA-PEG2-Pip-[NMe-Gly^11^]RM26; Pip: 4-amino-1-carboxymethyl-piperidine; PEG2: 8-Amino-3,6-dioxaoctanoic acid), ii. AU-RM26-M4 (DOTAGA-PEG2-Arg-Arg-Pip-[NMe-Gly^11^]RM26), iii. AU-SAR-M1 (DOTAGA-AMA-DIG-SAR; AMA: p-amino methylaniline; DIG: diglycolate) and iv. AU-SAR-M2 (DOTAGA-Arg-AMA-DIG-SAR), as shown in Fig. 1. Based on the increased in vivo stability and promising biodistribution profiles of the respective four [^111^In]In-radioligands, we opted to test the therapeutic potential of the corresponding [^177^Lu]Lu-labelled counterparts.

We herein present the preclinical evaluation of the above four [^177^Lu]Lu-labelled analogues, comprising head-to-head comparisons both in vitro (in prostate adenocarcinoma PC-3 cells) and in vivo in healthy and PC-3 xenograft bearing mice.

## Materials and methods

### Chemicals, reagents and radionuclides

Peptides were acquired form Pepmic Co. Ltd. (Suzhou, China). Trypsin––0.25% EDTA solution was obtained from Biochrom AG (Berlin, Germany); all other chemicals, reagents, growth media, supplements and consumables used were obtained from VWR International (Radnor, PA, USA). Entresto (24 mg/26 mg sacubitril/valsartan pills, Novartis, Basel, Switzerland) was sourced from a local pharmacy. All chemicals and reagents were of chemical grade and used without further purification unless mentioned otherwise.

Radiochemical grade Lu-177 (no carrier added) was purchased from ITM Medical Isotopes GmbH (Munich, Germany) in the form of [^177^Lu]LuCl_3_ in a 0.04 M HCl solution.

### Labelling and radiochemical analysis

For labelling the four analogues with Lu-177, in order to conduct in vitro and biodistribution studies, 5 µL of the corresponding peptide stock solution (1 mM in MQ water) were dissolved in 40 µL of NH_4_Ac (0.5 M, pH 5.5). Then, the following items were introduced in series, 20 µL ethanol, 10 µL ascorbic acid 0.1 M and 5 -20 µL of Lu-177 (50–75 MBq). The labelling solution was heated to 85 °C for 30 min.

For testing the in vivo stability, a slightly modified labelling protocol was followed. Thus, 10 µL of the peptide stock solution (10 nmol) under evaluation was labelled using 20 µL ethanol, 10 µL ascorbic acid 0.1 M, 80 µL ammonium acetate buffer (0.5 M, pH 5.5) and 113–135 µL of [^177^Lu]LuCl_3_ (229–244 MBq).

Radiochemical yields were assessed using instant thin layer chromatography (iTLC). Samples from the labelling solutions were applied on glass microfiber stripes, impregnated with silica gel (Agilent Technologies, Santa Clara, CA, USA) and they were developed using citric acid 0.2 M. Analysis of the percentage of activity on the application point (radiolabelled peptide) and the solvent front (free Lu-177) was done using a Cyclon Plus phosphor imager (PerkinElmer, Hägersten, Sweden).

Radiochemical purities were determined by reverse phase high performance liquid chromatography (RP-HPLC), using the following system: LaPrep Sigma HPLC LP1100 pump (Hitachi High-Tech Corporation, Hitachinaka, Ibaraki, Japan), 40D LWL UV-detector with a 4 µL flow cell (Knauer, Berlin, Germany), a Flow scan radioactivity detector with an FC-3300 NaI/PMT radioactivity probe (Eckert & Ziegler, Berlin, Germany), manual simple injector 7725i fitted with a 20 µL loop (IDEX Health & Science, LLC, CA, USA), Luna C18 column (5 µm, 100 Å, 150 × 4.6 mm from Phenomenex, Værløse, Denmark). The elution gradient went from 95% A/5% B to 40% A/60% B over 20 min (A: 0.1% v/v aqueous trifluoroacetic acid (TFA); B: 0.1% v/v TFA in acetonitrile).

In order to determine the LogD values for the four radioligands the following were introduced in an 1.5 mL Eppendorf tube in series: 500 µL of n-octanol, 500 µL of PBS and 30 pmol of the corresponding radiopeptide in 10 µL of PBS. The tubes were vortexed for 2 min and then centrifuged at 7000 g in order to speed up the separation of the layers. The n-octanol and PBS fractions were separated using a syringe and a needle and three 100 µL aliquots of each phase were collected and their activity contained was measured using a Wizard2^TM^ gamma-counter (PerkinElmer, Hägersten, Sweden).

### In vitro specificity, cellular uptake and receptor affinity

The GRPR-expressing human prostate cell line, PC-3, was purchased from ATCC (Manassas, VA, USA). Cells were cultivated using Roswell Park Memorial Institute (RPMI) 1640 medium containing L-glutamine, supplemented with 10% v/v FBS, penicillin (100 U/mL) and streptomycin (100 µg/mL). Cultures were kept at 37 °C in humidified environment with 5% CO_2_. Subculturing was performed twice per week, using a Trypsin–EDTA solution.

For in vitro specificity and cellular uptake experiments 8 × 10^5^ cells/well were seeded the day prior to the experiment in 6-well plates (specificity) or in 35 mm cell culture dishes (cellular uptake) and left to proliferate overnight.

To determine the specificity of the interaction between radioligands and GRPR, PC-3 cells seeded in 6-well plates were washed with fresh complete medium. Next, 1 mL fresh medium containing 1 nM of the labelled peptide under evaluation (total fraction) or the peptide with as × 1000-fold molar excess of NOTA-PEG2-RM26 (Varasteh et al. 2014) (blocked fraction) was introduced and the cells were left to incubate for 1 h at 37 °C. Cells were detached using a Trypsin–EDTA solution, cell suspensions were collected and their radioactivity content was measured using a gamma-counter. Experiments were performed in triplicate.

For estimation of cellular uptake over time, PC-3 cells seeded in 35 mm dishes were used. After washing with fresh media, the cells were left to incubate with 1 mL of media containing 1 nM of the radiolabelled peptide under evaluation at 37 °C. At 1, 4 and 24 h of incubation, the supernatant was removed and the dishes were placed over ice and washed with cold PBS. Next cells were treated twice with acid wash (glycine 0.2 M, NaCl 0.15 M, urea 4 M, pH 2, 4 °C) for 5 min and the supernatants were collected (membrane bound fraction). After a wash with PBS, cells were lysed using NaOH 1 M and lysates were collected (internalized fraction). Experiments were performed in triplicate and the activity content of the samples was measured on the gamma-counter.

Receptor affinity was measured using a LigandTracer yellow (Ridgeview Instruments AB, Uppsala, Sweden). For each measurement 3 million PC-3 cells were seeded in 100 mm dishes (Corning St. Oneonta, N.Y., USA) the day before the experiment. Three concentrations for each radioligand were tested (1, 3 and 5 nM) in order to determine the association values. After reaching plateau, the media containing the radioligands were removed, fresh complete media were introduced in the plates and the dissociation phase was measured overnight.

### In vivo studies

All animal experiments were conducted by authorized personnel in accredited facilities. Experimental protocols were approved by the Ethics Committee for Animal Research in Uppsala, Sweden (5.8.18-00473/2021; 2023/06/27).

### Stability in mouse blood circulation

To determine the in vivo stability of the four radioligands, four female NMRI mice per compound were used, divided into two groups: Control group and Entresto group (animals in this group were pre-treated 30 min prior to the injection with 12 mg Entresto pill per os, corresponding to 2.82 mg sacubitril). In short, each animal received a bolus iv injection of radioligand corresponding to 2 nmol of peptide and 46–50 MBq of Lu-177. After 5 min, animals were euthanized under anaesthesia and blood was collected directly from the heart, in pre-cooled Eppendorf tubes containing 40 µL of Na_2_-EDTA 20 mM solution. After mixing, blood samples were centrifuged at 2000 g and 4 °C for 10 min, using an Eppendorf Centrifuge 5430 R (Eppendorf Nordic A/S, Sverige Filial, Solna, Sweden). The plasma was collected and diluted with cold acetonitrile in one-to-one ratio and samples were centrifuged at 15,000 g and 4 °C for another 10 min. Supernatants were collected and concentrated by mild heating (45 °C), under a gentle stream of N_2_, until their volume was reduced to 50–100 µL. Samples were diluted with room-temperature PBS and 20 µL samples were analysed using reversed phase radio-HPLC. Results are given as percentage of intact peptide detected.

### Biodistribution in PC-3 xenograft bearing mice

The biodistribution profile for the four radioligands was determined in female Balb/c nu/nu mice. All the animals were subcutaneously implanted with a suspension of 8 × 10^6^ PC-3 cells in the right hind leg. After approximately 3–4 weeks, tumours were developed and animals were randomized in groups of four (with the exception of the Block groups comprising instead 3 animals). For each radioligand 3 groups were used, namely, 4 h, 23 h pi and 4 h Block. Each animal received 2.82 mg of sacubitril (in the form of 12 mg of an Entresto pill suspension) per os and after 30 min, a bolus iv injection of 100–110 kBq of [^177^Lu]Lu-peptide (corresponding to 100 pmol total peptide). Animals were euthanized at the predetermined time points and tissues of interest and xenografts were collected, weighted and their activity content was measured in the gamma counter. Animals in the Block groups received in addition a × 100 molar excess of NOTA-PEG2-RM26 (Varasteh et al. 2014) as a GRPR blocking agent to demonstrate in vivo specificity. Results are given as the average percentage of injected activity per gram of tissue (%IA/g) or else as (%IA) ± standard deviation (SD). Statistical analysis was performed using GraphPad Prism 10 and a two-way ANOVA with Tuckey’s post hock analysis. Area under curve for kidneys and tumours was estimated by fitting a one-phase decay model and calculating the area under the resulting equation.

## Results

### Labelling and radiochemical analysis

**Fig. 2:**
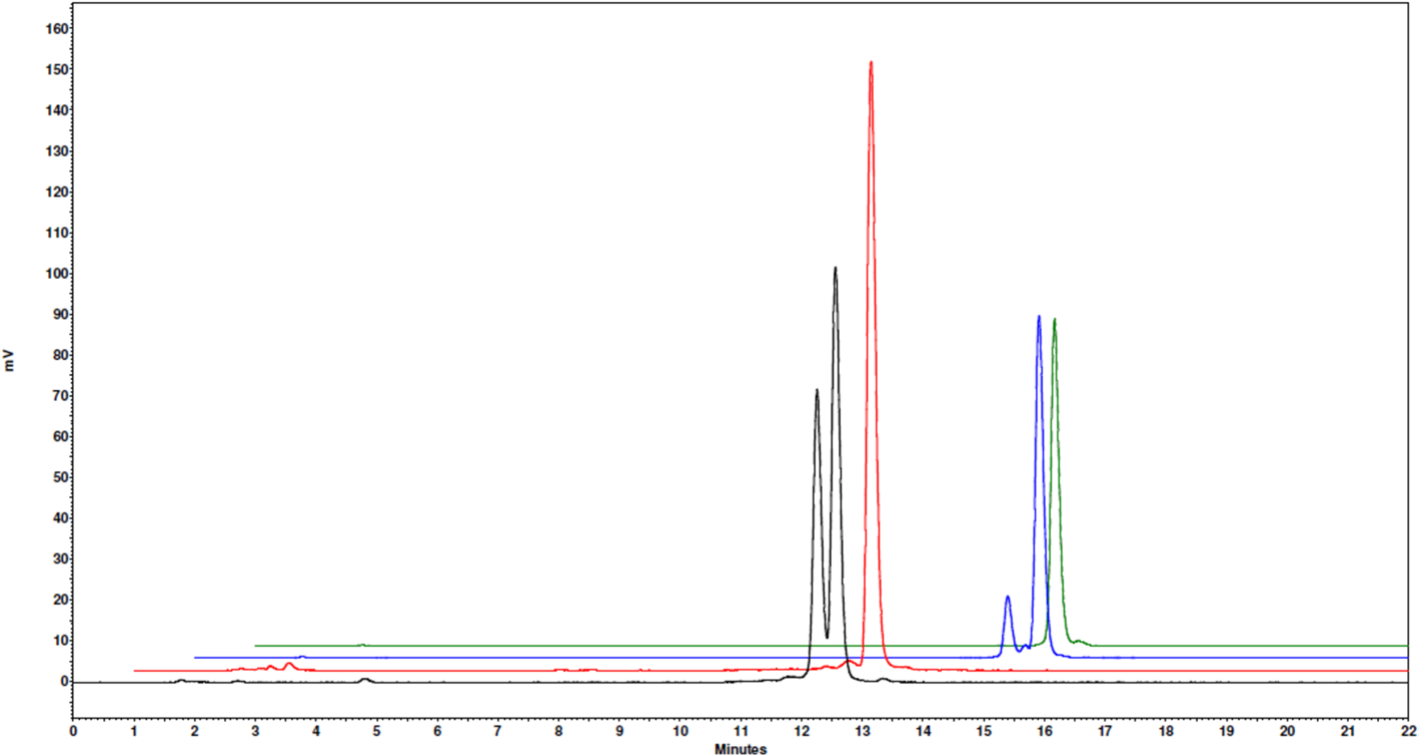
Representative radiochromatograms for the labelling solution of [^177^Lu]Lu-AU-RM26-M2 (black), [^177^Lu]Lu-AU-RM26-M4 (red), [^177^Lu]Lu-AU-SAR-M1 (blue) and [^177^Lu]Lu-AU-SAR-M2 (green)

All four peptides (Fig. 1) were successfully labelled with Lu-177 in high radiochemical yields (> 98%, based on iTLC analysis) and high radiochemical purities (> 95%, based on radio-HPLC analysis) at molar activities up to 24.4 MBq/nmol (Table 1 and Fig. 2). As can be seen in Fig. 2, there were two isomers present in the labelling solution for [^177^Lu]Lu-AU-RM26-M2 and [^177^Lu]Lu-AU-SAR-M4, which were considered to be biologically equivalent, based on previously reported results (Kanellopoulos et al. 2024; Obeid et al. 2024). Therefore, no separation of the two isomers was deemed necessary and accordingly, all radioligands underwent in vitro and in vivo testing without any further purification.

**Table 1:** Radiochemical data of [^177^Lu]Lu-AU-RM26-M2, [^177^Lu]Lu-AU-RM26-M4, [^177^Lu]Lu-AU-SAR-M1 and [^177^Lu]Lu-AU-SAR-M2

[^177^Lu]Lu-Radiopeptide	RCY (%) (iTLC)	RCP (%) (HPLC)	t_R_ (min) (HPLC)	LogD (n = 3)
AU-RM26-M2	> 98% (n = 10)	> 98% (n = 5)	12.3 – 12.6	− 2.54 ± 0.01
AU-RM26-M4	> 98% (n = 10)	> 98% (n = 5)	11.9	− 2.8 ± 0.03
AU-SAR-M1	> 98% (n = 10)	> 98% (n = 5)	13.2	− 2.61 ± 0.02
AU-SAR-M2	> 98% (n = 10)	> 98% (n = 5)	13.4 – 13.9	− 2.63 ± 0.02

### In vitro specificity and cellular uptake and receptor affinity

**Fig. 3:**
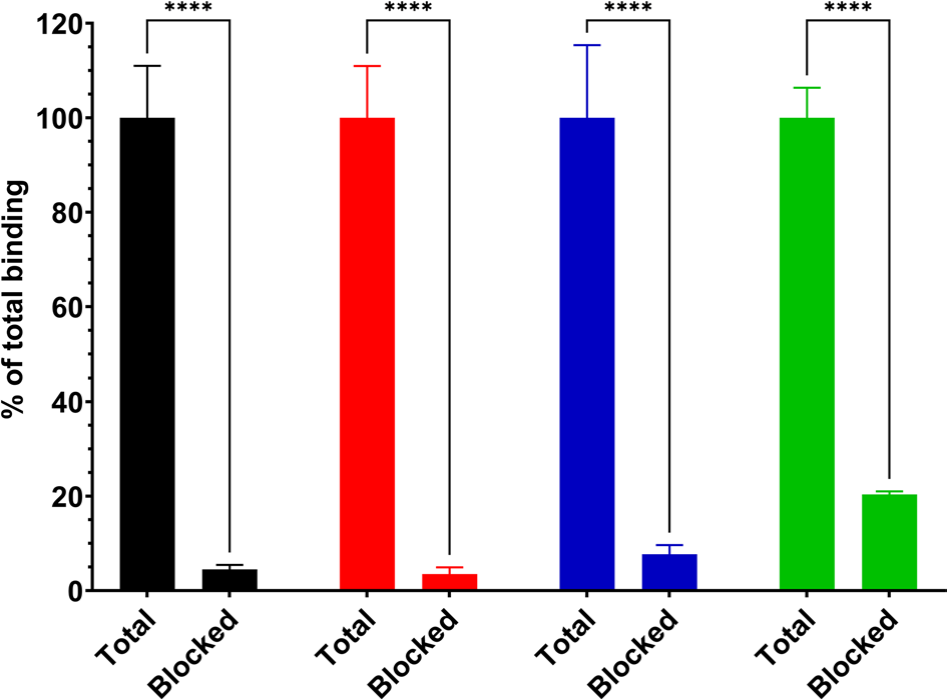
Specificity of [^177^Lu]Lu-AU-RM26-M2 (black), [^177^Lu]Lu-AU-RM26-M4 (red), [^177^Lu]Lu-AU-SAR-M1 (blue) and [^177^Lu]Lu-AU-SAR-M2 (green) in PC-3 cells

**Fig. 4:**
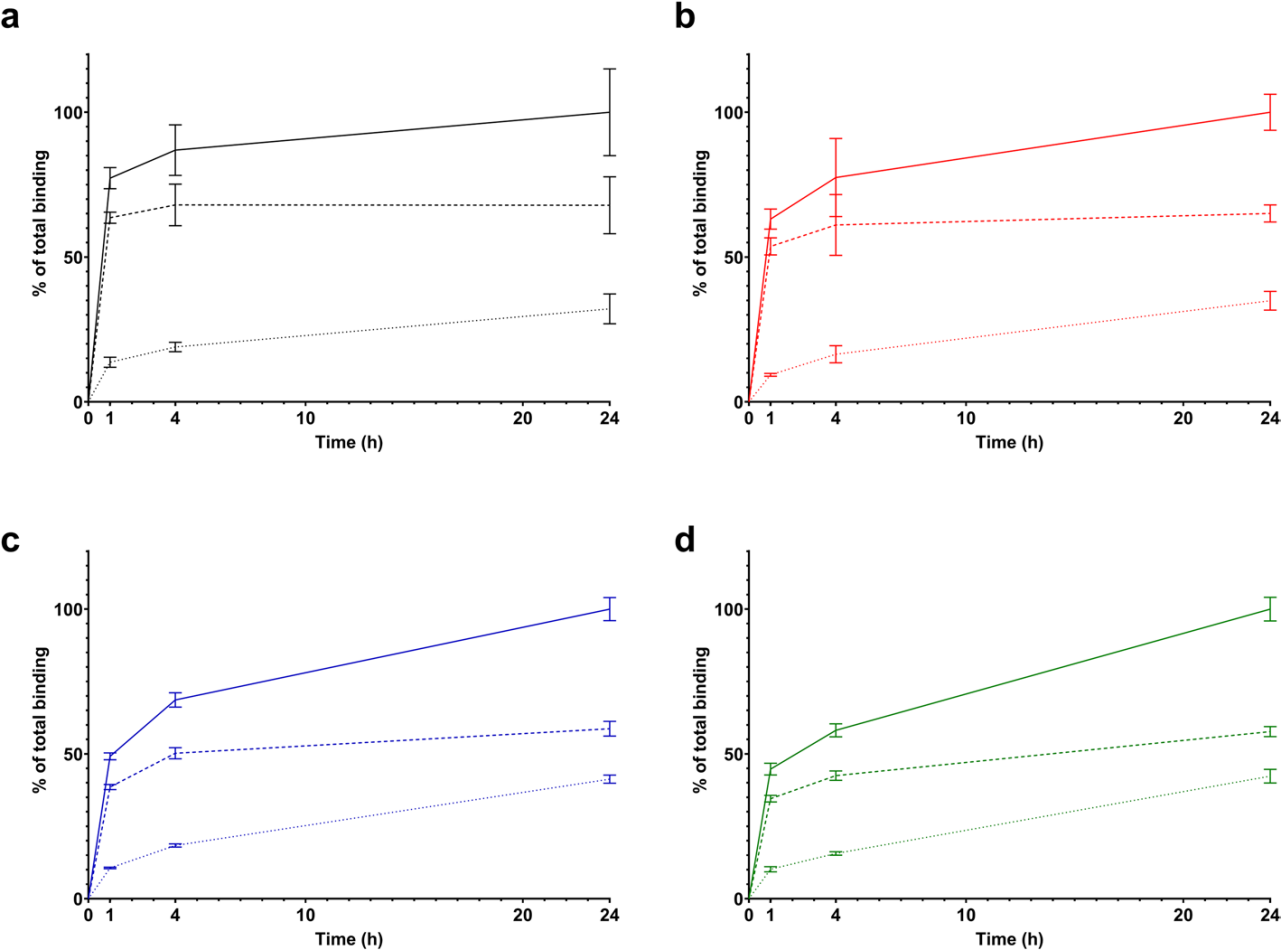
Cellular uptake of [^177^Lu]Lu-AU-RM26-M2 (black), [^177^Lu]Lu-AU-RM26-M4 (red), [^177^Lu]Lu-AU-SAR-M1 (blue) and [^177^Lu]Lu-AU-SAR-M2 (green) in PC-3 cells over time. Solid line corresponds to total binding, dashed lines correspond to membrane bound fraction and dotted lines to internalized fraction. Data are normalized to the highest total cellular uptake

All radiopeptides had high GRPR-mediated activity uptake in PC-3 cells in vitro, while displaying the typical profile of radioantagonists with the majority of the activity remaining on the cell membrane overtime, with a smaller portion of the cell-associated activity internalized after 24 h of continuous incubation (Figs. 3 and 4). At 1 h, [^177^Lu]Lu-AU-RM26-M2 had the highest internalization rate with 13.7 ± 1.7% of the cell-associated activity, followed by [^177^Lu]Lu-AU-SAR-M1 (10.6 ± 0.3%), [^177^Lu]Lu-AU-SAR-M2 (10.2 ± 0.9%) and [^177^Lu]Lu-AU-RM26-M4 (9.3 ± 0.5%). At 24 h, [^177^Lu]Lu-AU-SAR-M1 and [^177^Lu]Lu-AU-SAR-M2 had the highest internalized fraction (41.3 ± 1.4% and 42.3 ± 2.4%, respectively), while [^177^Lu]Lu-AU-RM26-M2 and [^177^Lu]Lu-AU-RM26-M4 followed with 32.1 ± 5.2% and 34.9 ± 3.3% of total cell-bound activity, respectively.

When the affinity for GRPR was assessed in living PC-3 cells, a 1–2 model was used since previous reports have suggested that there are two types of interaction between GRPR and the analogues, a dominant high affinity interaction (K_D1_) and a second one with lower affinity (K_D2_) (Xu et al. 2013). All labelled peptides displayed sub-nanomolar K_D1_ values (Table 2), with K_D2_ values ranging from nanomolar down to sub-nanomolar. Among these radioligands, [^177^Lu]Lu-AU-SAR-M2 (K_D_: 7.08 × 10^–12^ M) displayed the highest receptor affinity. These values were a bit lower than the ones reported for their [^111^In]In-labelled counterparts previously reported (Kanellopoulos et al. 2024; Obeid et al. 2024).

**Table 2:** Kinetic and affinity data for the four [^177^Lu]Lu-labelled GRPR-antagonists, based on LigandTracer graph analysis

[^177^Lu]Lu-	k_a1_ (M^−1^ × s^−1^)	k_d1_ (s^−1^)	K_D1_ (M)	k_a2_ (M^−1^ × s^−1^)	k_d2_ (s^−1^)	K_D2_ (M)
AU-RM26-M2	1.84 × 10^5^	3.53 × 10^–5^	19.2 × 10^–10^	1.08 × 10^6^	5.94 × 10^–2^	5.52 × 10^–8^
AU-RM26-M4	5.52 × 10^5^	8.73 × 10^–6^	1.58 × 10^–11^	1.31 × 10^7^	5.34 × 10^–1^	4.09 × 10^–8^
AU-SAR-M1	6.57 × 10^4^	1.76 × 10^–6^	2.67 × 10^–11^	6.14 × 10^5^	7.43 × 10^–4^	1.21 × 10^–9^
AU-SAR-M2*	9.09 × 10^4^	6.44 × 10^–7^	7.08 × 10^–12^	–	–	–

^*^Due to its very high affinity the evaluation with the 1:2 model gave results out of the range of trustworthy values, thus a 1:1 model was used to estimate the affinity

### Stability in mouse blood circulation

**Fig. 5:**
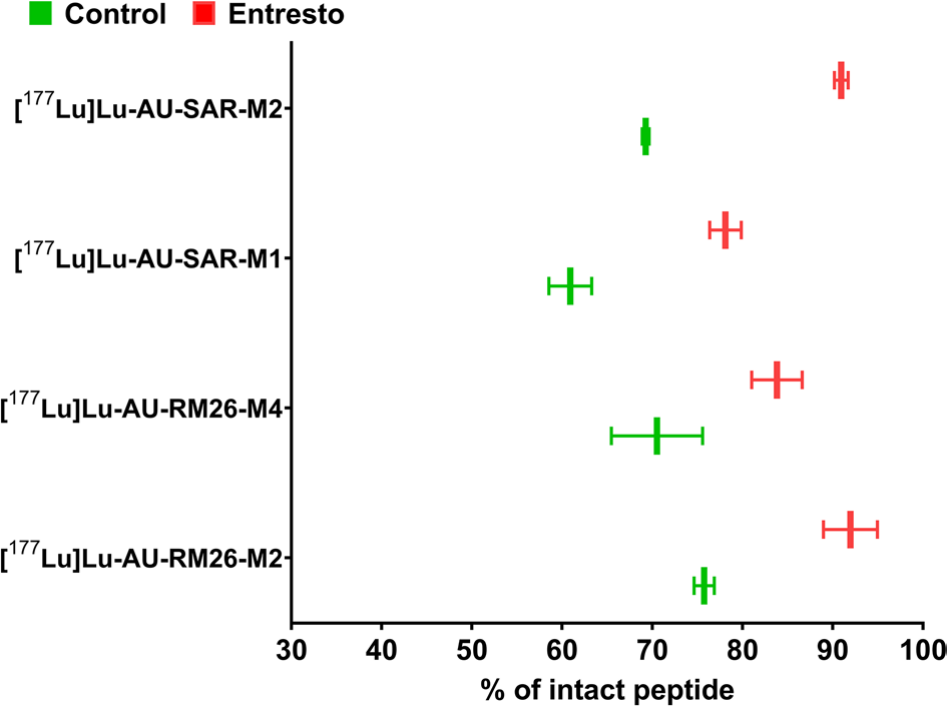
In vivo stability, as determined by radio-HPLC analysis, of [^177^Lu]Lu-AU-RM26-M2, [^177^Lu]Lu-AU-RM26-M4, [^177^Lu]Lu-AU-SAR-M1 and [^177^Lu]Lu-AU-SAR-M2 in NMRI mice peripheral blood. Control (green): untreated groups; Entresto (red): groups pre-treated with Entresto

The four radioligands demonstrated good in vivo stability in blood circulation (Fig. 5, Table S1 and Figures S1-S8). [^177^Lu]Lu-AU-RM26-M2 and [^177^Lu]Lu-AU-RM26-M4 displayed the highest in vivo stability, with 76% and 71% of intact peptide detected in blood plasma 5 min post injection (pi), respectively. The [^177^Lu]Lu-AU-SAR-M1 and [^177^Lu]Lu-AU-SAR-M2 demonstrated a slightly lower stability. Although all four analogues were fairly stable in the blood stream, their stability could be further increased by pre-treatment of the animals with Entresto. Thus, the respective values reached: [^177^Lu]Lu-AU-RM26-M2—92%, [^177^Lu]Lu-AU-RM26-M4—84%, [^177^Lu]Lu-AU-SAR-M1—78% and [^177^Lu]Lu-AU-SAR-M2—91%. The substantial increase in their stability induced by Entresto (18–32%), strongly supports the involvement of NEP in their degradation in circulation.

### Biodistribution in PC-3 xenograft bearing mice

**Fig. 6:**
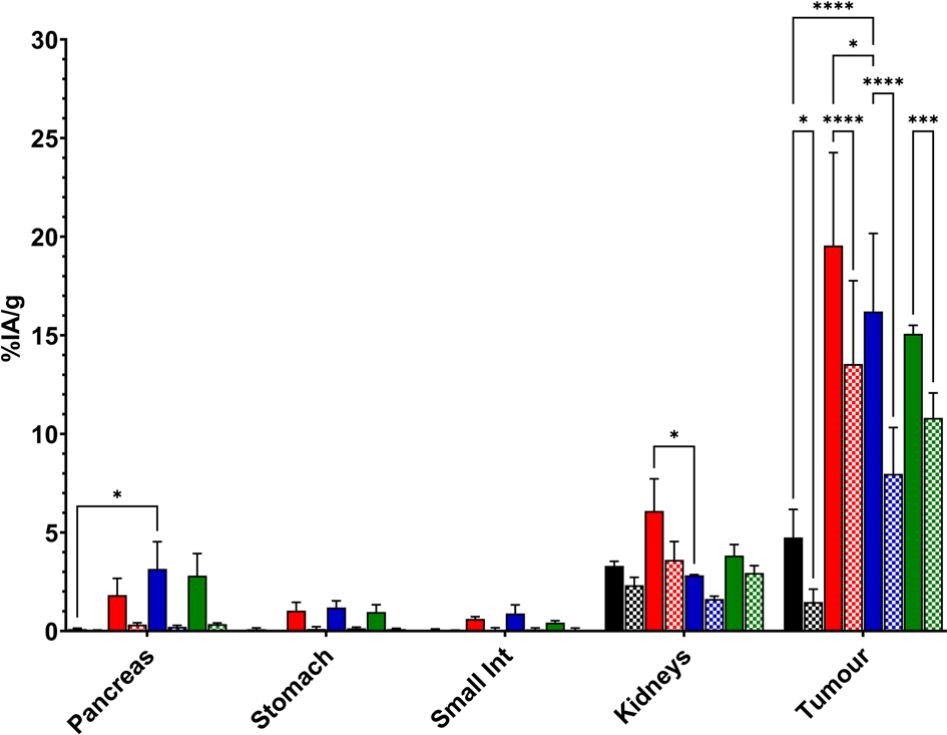
Graphical representation of selected biodistribution data of [^177^Lu]Lu-AU-RM26-M2 (black), [^177^Lu]Lu-AU-RM26-M4 (red), [^177^Lu]Lu-AU-SAR-M1 (blue) and [^177^Lu]Lu-AU-SAR-M2 (green) in PC-3 xenograft bearing mice as average %IA/g ± sd, at 4 h (solid bars) and 23 h pi (checkered bars). Small Int: small intestines, Tumour: PC-3 xenograft. Statistically significant differences are denoted with *: *p* < 0.05, **: *p* < 0.01, ***: *p* < 0.001 and ****: *p* < 0.0001 in the graph

The biodistribution profiles of [^177^Lu]Lu-AU-RM26-M2, [^177^Lu]Lu-AU-RM26-M4, [^177^Lu]Lu-AU-SAR-M1 and [^177^Lu]Lu-AU-SAR-M2 were determined in nu/nu mice bearing PC-3 xenografts at 4 h and 23 h pi; mice were pre-treated with Entresto (12 mg) to maximize their in vivo stability. All radioligands displayed rapid clearance from healthy tissues as early as 4 h pi (Fig. 6 and Tables S2 and S3). The organs/tissues with the highest activity accumulation at 4 h pi were the kidneys (6–3%IA/g) and the GRPR-rich pancreas (3–2%IA/g; whereas for [^177^Lu]Lu-AU-RM26-M2 – 0.1 ± 0.05%IA/g) and to a lesser extent the stomach (< 2%IA/g) and small intestines (< 1.2%IA/g) (Baratto et al. 2020; Sano et al. 2004; Xiao et al. 2001).

The highest tumour activity uptake at 4 h pi was observed for [^177^Lu]Lu-AU-RM26-M4 (15 ± 0.4%IA/g). At 23 h pi, the radioligands were washed out from the majority of the healthy tissues (< 0.5%IA/g for all organs/tissues) and to a great extent from kidneys ([^177^Lu]Lu-AU-RM26-M2–30.3%; [^177^Lu]Lu-AU-RM26-M4–40%; [^177^Lu]Lu-AU-SAR-M1–44.5%; [^177^Lu]Lu-AU-SAR-M4–21.1% decreases). Tumour uptake was variable across analogues at 4 h pi ([^177^Lu]Lu-AU-RM26-M2: 1.5 ± 0.05%IA/g; [^177^Lu]Lu-AU-RM26-M4: 15 ± 4%IA/g, [^177^Lu]Lu-AU-SAR-M1: 8 ± 2%IA/g; [^177^Lu]Lu-AU-SAR-M2: 11 ± 1%IA/g) and GRPR-specific, since co-injection of a 100 × molar excess of NOTA-PEG2-RM26 led to a substantial decrease of tumour uptake (≥ 89% decrease in activity uptake; Tables S1 and S2). Between the 4 h and 23 h pi intervals, the tumour values dropped, but three out of 4 analogues displayed quite pronounced tumour retention. Specifically, the activity uptake at 23 h pi was about 75% for [^177^Lu]Lu-AU-RM26-M4 and [^177^Lu]Lu-AU-SAR-M2, 50% for [^177^Lu]Lu-AU-SAR-M1, and 30% for [^177^Lu]Lu-AU-RM26-M2 of the activity uptake at 4 h pi.

**Fig. 7:**
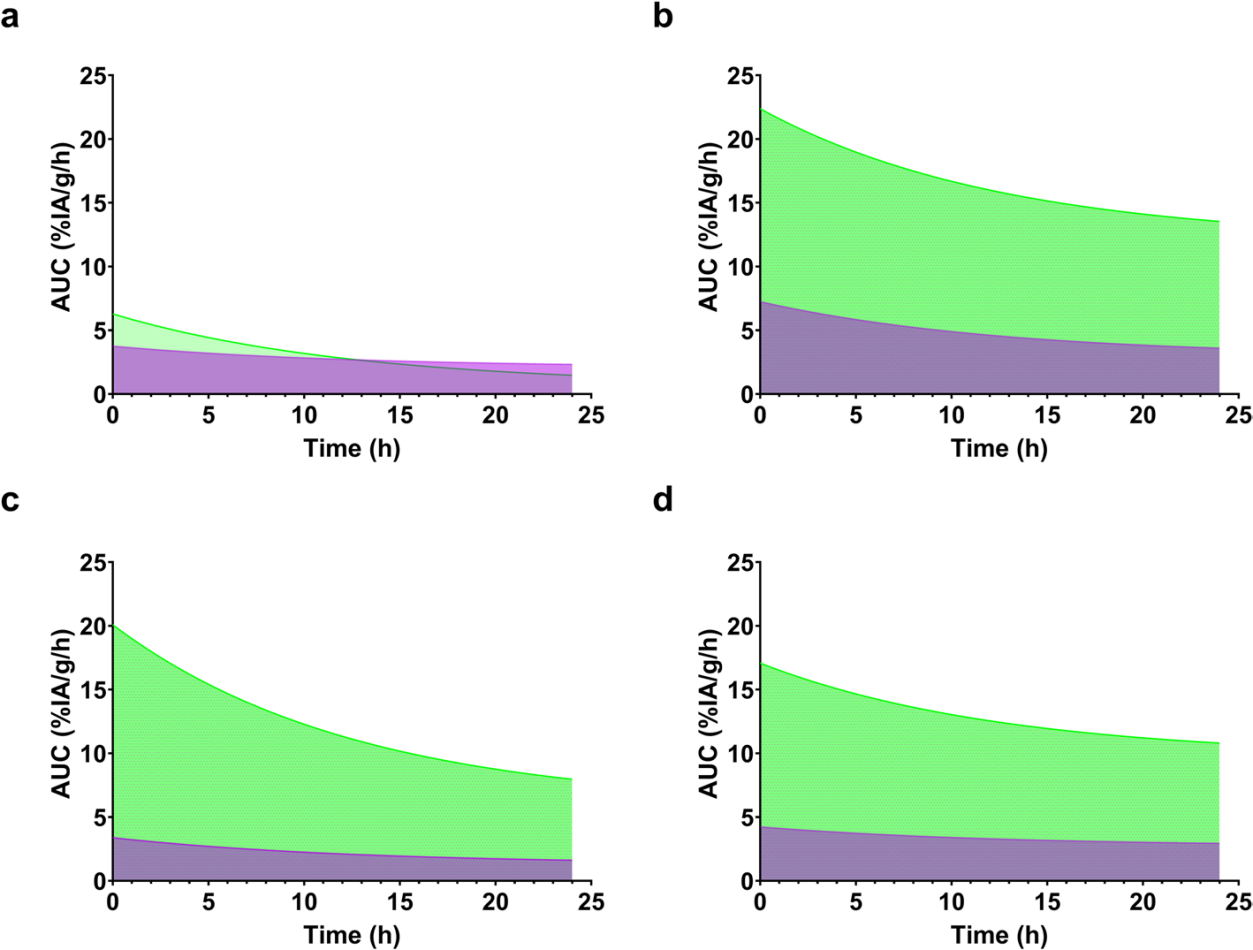
Estimated AUC for kidneys (purple) and tumours (green) for [^177^Lu]Lu-AU-RM26-M2 (**A**), [^177^Lu]Lu-AU-RM26-M4 (**B**), [^177^Lu]Lu-AU-SAR-M1 (**C**) and [^177^Lu]Lu-AU-SAR-M2 (**D**)

This is particularly relevant for tumour vs. kidney values, as clearly apparent from the area under the curve (AUC) calculated for kidneys and tumours for all four analogues (Fig. 7 and Table S4). These values represent a good measure of their residence time in these tissues. Their potential therapeutic efficacy could be estimated by calculating the ratio of AUCs between tumour/kidneys for each analogue. Based on the latter ratios, [^177^Lu]Lu-AU-SAR-M2 outperformed the rest of the analogues, achieving the best Tumour-to-Kidneys AUC ratio. The latter was estimated to be 1.4-fold higher than [^177^Lu]Lu-AU-SAR-M1 and 1.6-fold higher than [^177^Lu]Lu-AU-RM26-M4.

## Discussion

The overexpression of GRPR in various human malignancies (e.g. prostate cancer, breast cancer, GIST, gastrinomas, small cell lung cancer) with minimal expression in healthy tissues (except for the pancreas and the GIT), makes the GRPR a very appealing biomolecular target both for imaging and peptide receptor radionuclide therapy (PRRT). In addition, peptides owing to the very rapid kinetics, high specificity, low immunogenicity, low production cost and chemical modification versatility represent an excellent platform for the design of potent target-specific radiopharmaceuticals. Despite these advantages, peptide-based radioligands are prone to fast degradation in the biological milieu by proteolytic enzymes, known as proteases or peptidases. The latter are present in the blood plasma or serum, and can be also found anchored on epithelial cells in the vasculature and major organs (e.g. NEP) (Roques et al. 1993). Circumventing the major drawback of peptide-based drugs, including radiopharmaceuticals, requires a series of trials in order to pin-point the appropriate structural interventions that can increase the in vivo stability without compromising receptor affinity or pharmacokinetics (Vlieghe et al. 2010). The current study is part of a series of studies aiming to investigate such structural interventions and their impact upon the performance of GRPR-targeting radiotheranostics (D’Onofrio et al. 2023; Mitran et al. 2020; Nock et al. 2023).

Previous studies on a number of [^111^In]In-DOTAGA modified GRPR-antagonists revealed the four best performing analogues (Fig. 1), whereby positive charge(s) in the linkers led to enhanced GRPR-affinities and improved pharmacokinetics (Abouzayed et al. 2023; Kanellopoulos et al. 2024; Obeid et al. 2024). Furthermore, all analogues were NMe-Gly^11^ substituted, showing higher resistance to peptidases, thus increasing the effective amount of the intact radioligand reaching the tumour sites (Abouzayed et al. 2023). These findings were in line with previous reports, underlining the importance of in vivo stability, especially in the initial couple of minutes after injection required for the peptide radioligand to reache tumour-sites (Kanellopoulos et al. 2020; Nock et al. 2014; Shipp et al. 1991).

In continuation of these investigations, AU-RM26-M2, AU-RM26-M4, AU-SAR-M1 and AU-SAR-M2 were successfully labelled with Lu-177 in high radiochemical yields and purities in this study (Table 1). The high molar activities and radiochemical purities achieved ensure an easier translation into clinics without the need of time consuming and complex purification steps. In PC-3 cells, the four radioligands displayed high cellular and GRPR-specific uptake, while retaining the typical profile of radioantagonists with the bulk of the radioactivity remaining on the cell membrane.

[^177^Lu]Lu-AU-RM26-M2, [^177^Lu]Lu AU-RM26-M4, [^177^Lu]Lu AU-SAR-M1 and [^177^Lu]Lu AU-SAR-M2 showed high affinity for GRPR according to the results from receptor––ligand kinetics analysed with LigandTracer. K_D_ values for all radiopeptides were found in the sub-nanomolar range with [^177^Lu]Lu-AU-SAR-M2 displaying the highest receptor affinity (Table 2), owing to a great part to the Arg-AMA-DIG linker. This linker combines an extra positive charge in combination with the AMA-DIG sequence, shown to increase GRPR-affinity (Mitran et al. 2020; Wang et al. 2024). These results corroborate the results reported for the respective [^111^In]In-radioligand counterparts (Kanellopoulos et al. 2024; Obeid et al. 2024).

The four [^177^Lu]Lu-radioligands displayed good resistance to peptidases in the blood stream, with > 60% detected intact 5 min pi. [^177^Lu]Lu-AU-RM26-M2 turned out to be the most stable one. Significant stability increases were achieved by pre-treatment of mice with Entresto (Gu et al. 2010; Kanellopoulos et al. 2020; McMurray et al. 2014; Schiering et al. 2016). The administration of Entresto increased 1.2–1.3-fold the percentage of the peptide remaining intact (Table S4), in agreement with previous reports (Kanellopoulos et al. 2020; Mitran et al. 2019; Rottenburger et al. 2024). In order to compare the maximum potential of the new agents, their biodistribution profiles were evaluated during Entresto pre-treatment, i.e., when equally high stability could be reached in circulation.

In a previous peptide mass escalation study, [^177^Lu]Lu-DOTAGA-PEG2-RM26 (Mitran et al. 2019), could achieve better tumour penetration and better clearance from the background tissues at a peptide mass of 250 pmol, while further increase of injected peptide mass led to unfavourably increase of saturation of tumour-situated receptors and, hence decrease of tumour uptake. In order to advantageously counterbalance the effects of low vs. high peptide mass, 100 pmol of peptide were injected in the present work in combination with Entresto pre-treatment. As a result, the four [^177^Lu]Lu-radioligands displayed very low activity uptake in the majority of healthy tissues at 4 h pi. The activity uptake was somewhat higher for the physiologically GRPR-expressing organs, with the highest values observed for pancreas, which eventually was washed out as well by 23 h pi. The kidneys turned out to be the dose limiting organ, showing the highest uptake and retention overtime. This information will be taken into account and is crucial for the design of the PRRT scheduled to be conducted next. The kidney activity uptake of the four radioligands tested herein decreased by 43% between 4 and 23 h pi.

To better evaluate these findings, the AUC for the kidneys and tumours were calculated, as a means to estimate their residence times in these organs and thus, predict their therapeutic efficacy. As it can be seen in Table S4, the highest tumour AUC was found for [^177^Lu]Lu-AU-RM26-M4 (X: Arg-Arg-Pip), which was 1.3-fold higher than for [^177^Lu]Lu-AU-SAR-M1 (X: AMA-DIG) and [^177^Lu]Lu-AU-SAR-M2 (Arg-AMA-DIG), and over fivefold higher than for [^177^Lu]Lu-AU-RM26-M2 (PEG2-Pip). In the kidneys, the longer residence was also found for [^177^Lu]Lu-AU-RM26-M4, 1.7–twofold over the other three tested radiopeptides. When the tumour-to kidneys ratio of the AUCs was estimated, as a measure of therapeutic index, the ranking changed as follows: [^177^Lu]Lu-AU-SAR-M1 > [^177^Lu]Lu-AU-SAR-M2 > [^177^Lu]Lu-AU-RM26-M4 > [^177^Lu]Lu-AU-RM26-M2. Thus, [^177^Lu]Lu-AU-SAR-M1 turned out to display better prospects as a radiotherapeutic agent, although ranking second in tumour uptake. Due to its lower kidney retention, a lower renal toxicity is expected. Eventually, [^177^Lu]Lu-AU-SAR-M1 was deemed the most promising analogue for further evaluation in dosimetry and therapy studies. This data, is in good agreement with the results of the biodistribution studies for the corresponding [^111^In]In-radioligands (Kanellopoulos et al. 2024; Obeid et al. 2024).

From the biodistribution data a few significant conclusions could be drawn. Firstly, despite the very high stability induced after pre-treatment of the animals with Entresto, [^177^Lu]Lu-AU-RM26-M2 ended up displaying the lowest tumour uptake among the four analogues. This finding is most likely indicative of its lower affinity for GRPR (Table 2), since [^177^Lu]Lu-AU-RM26-M2 displayed a K_D1_ at least 2-orders of magnitude higher than the rest of the analogues. Secondly, the introduction of more positive charges in the N-terminus in the linker resulted in higher affinity for the GRPR, in agreement with previous reports (Mitran et al. 2020). Thirdly, the increase in affinity as a result of the incorporation of the Arg residues in the linkers, did not always result in better tumour targeting, for example in comparison between [^177^Lu]Lu-AU-SAR-M1 (X: AMA-DIG) and [^177^Lu]Lu-AU-SAR-M2 (X: Arg-AMA-DIG) there is no statistical difference for the activity uptake in tumours either at 4 h or 24 h pi (Fig. 6). Lastly, the introduction of the Arg residues in the linkers, while resulting in better activity retention in tumours (due to the increase of GRPR-affinity), inadvertently led to increased retention in the kidneys.

At that point it would be interesting to compare how the [^177^Lu]Lu-AU-SAR-M1 + Entresto regimen fares compared with other Lu-177-based GRPR-antagonists, such as [^177^Lu]Lu-NeoBomb1 or [^177^Lu]Lu-RM2, both already tested in patients.

In comparison with [^177^Lu]Lu-NeoBomb1 (Dalm et al. 2017; Nock et al. 2017), [^177^Lu]Lu-AU-SAR-M1 utilizes a different chelator (DOTAGA for AU-SAR-M1 and DOTA for NeoBomb1). In addition, the two peptide analogues have different C-termini, with [^177^Lu]Lu-AU-SAR-M1 ending in the sequence His-Leu-NHEt, while [^177^Lu]Lu-NeoBomb1 at His-NHCH[(CH_2_-CH(CH_3_)_2_]_2_. As it is reported, [^177^Lu]Lu-NeoBomb1 is more stable in mice circulation, with 95% intact peptide detected at 5 min pi (Nock et al. 2017), in comparison with 61% found for [^177^Lu]Lu-AU-SAR-M1 (78% under the influence of Entresto). When tested in PC-3 xenograft bearing mice, [^177^Lu]Lu-NeoBomb1 displayed better tumour activity uptake with the higher mass of injected peptide (200 pmol – 1 MBq) vs. the lower one (10 pmol – 1 MBq), further supporting our decision for injection of 100 pmol of peptide (Dalm et al. 2017). At 4 h pi, tumour values were 17.9 ± 3.3%IA/g, which are on the same level with the values we present for [^177^Lu]Lu-AU-SAR-M1 in combination with Entresto pre-treatment (16 ± 4%IA/g) for the same time point. The same trend is shown for the kidneys as well (2.5% for [^177^Lu]Lu-NeoBomb1 and 2.83% for [^177^Lu]Lu-AU-SAR-M1). The difference between the two radioligand regimens is at the values observed for pancreas. The latter could be tentatively attributed to variable interspecies GRPR-affinities displayed by different families of GRPR-antagonists (Maina et al. 2005). After overcoming the stability differences in circulation using Entresto, it appears that the overall pharmacokinetics of [^177^Lu]Lu-AU-SAR-M1 are on par with the [^177^Lu]Lu-NeoBomb1.

For [^177^Lu]Lu-RM2 (Dumont et al. 2013), the reported dose per animal was 250 pmol peptide corresponding to 4.63 MBq. Despite the lower tumour uptake reported for [^177^Lu]Lu-RM2 (10.97 ± 0.99%IA/g at 4 h pi) in comparison with [^177^Lu]Lu-AU-SAR-M1 (in the Entresto-treated animals), the retention follows the same pattern with approximately 50% of the activity remaining in the tumours at 24 h pi. Values for kidneys were on the same level as for [^177^Lu]Lu-NeoBomb1 and [^177^Lu]Lu-AU-SAR-M1, but surprisingly [^177^Lu]Lu-RM2 had quite lower activity uptake in pancreas (0.70 ± 0.12%IA/g at 4 h pi).

Biological differences between the strains of animals used, possible variations across PC-3 cell batches, the different molar activities used for the in vivo evaluation of [^177^Lu]Lu-AU-SAR-M1, [^177^Lu]Lu-NeoBomb1 and [^177^Lu]Lu-RM2, the in situ stabilization of [^177^Lu]Lu-AU-SAR-M1 with the use of Entresto and the distinct interspecies GRPR-affinities that different families of antagonists may exhibit (Maina et al. 2005) need to be carefully considered before reaching into further reliable conclusions. Nevertheless, from the above comparison it seems that the combination of [^177^Lu]Lu-AU-SAR-M1 with Entresto pre-treatment, is on equal footing in terms of expected therapeutic efficacy as the two other well-established radioantagonists. This finding provides the opportunity to provide a “second life” to radiopharmaceuticals with good overall pharmacokinetics but prone to degradation in vivo allowing them to nevertheless show high therapeutic efficacy prospects. Currently ongoing therapeutic studies will finally establish the validity of this hypothesis.

## Conclusions

Among the four GRPR-radioantagonist tested in this study, [^177^Lu]Lu-Au-SAR-M1 had a superior overall performance. It displayed 1.4 times better tumour-to-kidney ratio for the estimated AUC than the next candidate. This finding is indicative of its potential as a radiotherapeutic agent against GRPR-expressing cancer.

## Supplementary Information

Supplementary file 1.

## Data Availability

All data generated or analyzed during this study are included in this published article and its supplementary information files.

## Abbreviations

RM2: DOTA-4-amino-1-carboxymethyl-piperidine-DPhe-Gln-Trp-Ala-Val-Gly-His-Sta-Leu-NH_2_
LF1: AAZTA^5^-Pip-DPhe-Gln-Trp-Ala-Val-Gly-His-Sta-Leu-NH_2_ (AAZTA^5^: 6-[Bis (carboxymethyl)amino]-1,4-bis (carboyxmethyl)-6-methyl-1,4-diazepane)
LE1: SARC-PEG4-DPhe-Gln-Trp-Ala-Val-Gly-His-Sta-Leu-NH_2_ (SARC: sarcophagine, 5-(8-methyl-3,6,10,13,16,19-hexaaza-bicyclo[6.6.6]icosan-1-ylamino)-5-oxopentanoic acid) )
DOTAM-GRPR1: DOTAM-βAla-βAla-DPhe-Gln-Trp-Ala-Val-Gly-His-Sta-Leu-NH_2_ (DOTAM: 1,4,7,10-tetrakis(carbamoylmethyl)-1,4,7,10-tetraazacyclododecane)
AMTG: DOTA-Pip-DPhe-Gln-L-α-Methyl-Trp-Ala-Val-Gly-His-Sta-Leu-NH_2_
NeoBomb1: DOTA-AMA-DIG-DPhe-Gln-Trp-Ala-Val-Gly-His-NHCH[(CH_2_-CH(CH_3_)_2_]_2_
SB3: DOTA-AMA-DIG-DPhe-Gln-Trp-Ala-Val-Gly-His-Leu-NHEt
