# Supplementary material for: RM2 and DB15 analogues bearing [177Lu]Lu-DOTAGA via different linkers, as radiotherapeutics: a head-to-head comparative study
Source: EJNMMI Radiopharm Chem. 2025 Jul 31;10:50. doi: 10.1186/s41181-025-00374-3 (PMC12314183; doi:10.1186/s41181-025-00374-3)
Supplement: Supplementary file 1 [file 41181_2025_374_MOESM1_ESM.docx]

# Supplementary material

# Title

RM2 and DB15 analogues bearing [^177^Lu]Lu-DOTAGA via different linkers, as radiotherapeutics: A head-to-head comparative study

# Authors

Panagiotis Kanellopoulos^1*^, Athanasios Bitzios^1^, Ivan Zelepukin^1^, Ekaterina Bezverkhniaia^1^, Theodosia Maina^2^, Berthold A. Nock^2^, Vladimir Tolmachev^3^, Anna Orlova^1,4^

1. Department of Medicinal Chemistry, Uppsala University, Uppsala 751 23, Sweden
2. Molecular Radiopharmacy, INRaSTES, NCSR “Demokritos”, 15341 Athens, Greece
3. Department of Immunology, Genetics and Pathology, Uppsala University, Uppsala 751 83, Sweden.
4. Science for Life Laboratory, Uppsala University, Uppsala 752 37, Sweden

* Corresponding author

**Corresponding Author**

Panagiotis Kanellopoulos − Department of Medicinal Chemistry, Uppsala University, Uppsala 751 23, Sweden; orcid.org/0000-0002-0617-3936; Email: Panagiotis.kanellopoulos@ilk.uu.se

**Table S1.** Percentage of intact peptide detected by radio-HPLC analyses in the mice circulation without (control) or after treatment with Entresto. Results are given as mean with the two recorded values for each group reported in the parenthesis.

|  | **Control** | **Entresto** |
| --- | --- | --- |
| **Compound** | **Mean [n1 – n2]** | **Mean [n1 – n2]** |
| **[^177^Lu]Lu-AU-RM26-M2** | 76% [77% - 75%] | 92% [89% - 95%] |
| **[^177^Lu]Lu-AU-RM26-M4** | 71% [76% - 65%] | 84% [87% - 81%] |
| **[^177^Lu]Lu-AU-SAR-M1** | 61% [59% - 63%] | 78% [80% - 76%] |
| **[^177^Lu]Lu-AU-SAR-M2** | 69% [69% - 70%] | 91% [90% - 92%] |


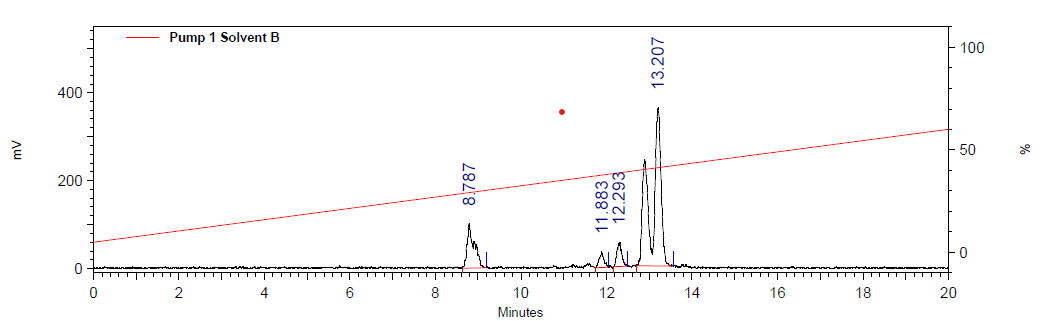


***Figure S1****. Representative radiochromatogram of [^177^Lu]Lu-AU-RM26-M2 (control) in mice peripheral blood at 5 min pi.*


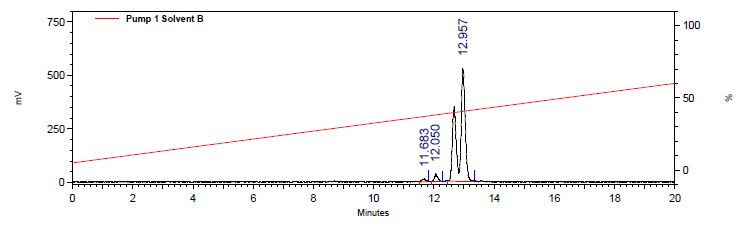


***Figure S2****. Representative radiochromatogram of [^177^Lu]Lu-AU-RM26-M2 (Entresto) in mice peripheral blood at 5 min pi during in situ NEP-inhibition.*


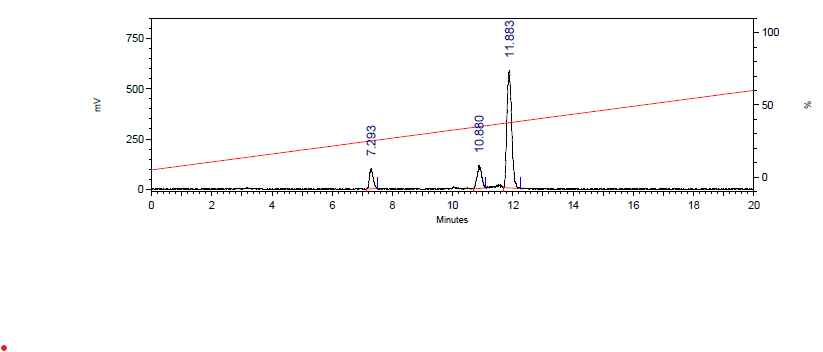


***Figure S3****. Representative radiochromatogram of [^177^Lu]Lu-AU-RM26-M4 (control) in mice peripheral blood at 5 min pi.*


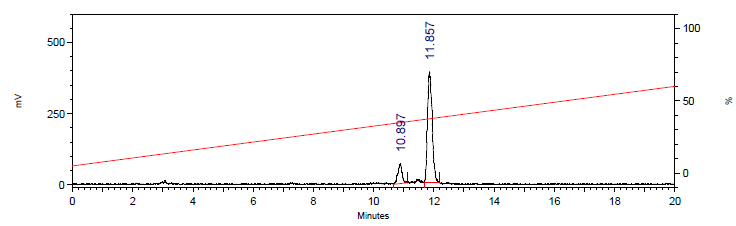


***Figure S4****. Representative radiochromatogram of [^177^Lu]Lu-AU-RM26-M4 (Entresto) in mice peripheral blood at 5 min pi during in situ NEP-inhibition*


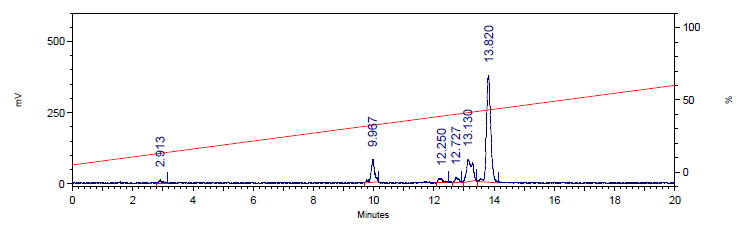


***Figure S5****. Representative radiochromatogram of [^177^Lu]Lu-AU-SAR-M1 (control) in mice peripheral blood at 5 min pi*.


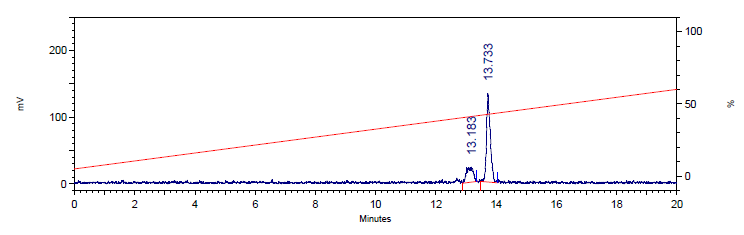


***Figure S6****. Representative radiochromatogram of [^177^Lu]Lu-AU-SAR-M1 (Entresto) in mice peripheral blood at 5 min pi during in situ NEP-inhibition*


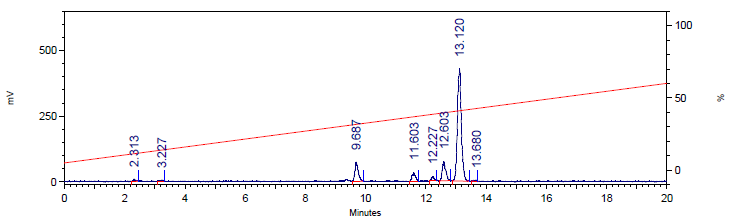


***Figure S7.*** *Representative radiochromatogram of [^177^Lu]Lu-AU-SAR-M2 (control) in mice peripheral blood at 5 min pi.*


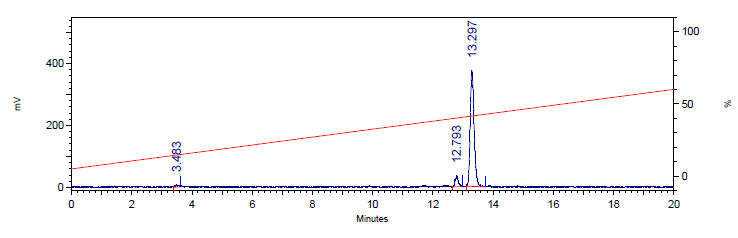


***Figure S8****. Representative radiochromatogram of [^177^Lu]Lu-AU-SAR-M2 (Entresto) in mice peripheral blood at 5 min pi during in situ NEP-inhibition*

**Table S2**. Biodistribution profile of [^177^Lu]Lu-AU-RM26-M2 and [^177^Lu]Lu-AU-RM26-M4 in PC-3 xenograft bearing mice at 4 h and 23 h pi. All animals were pre-treated with Entresto as a means of in situ inhibition of NEP. Block groups received in addition a 100× molar excess of GRPR-blocking agent. Results are given as percentage of injected activity per gram of tissue (%IA/g) with the exception of carcass and gastrointestinal track (GI) which are given as percentage of injected activity (%IA).

|  | **[^177^Lu]Lu-AU-RM26-M2** | | | **[^177^Lu]Lu-AU-RM26-M4** | | |
| --- | --- | --- | --- | --- | --- | --- |
| **Organs** | **4 h Block** | **4 h** | **23 h** | **4 h Block** | **4 h** | **23 h** |
| **Blood** | 0.01 ± 0.00 | 0.02 ± 0.01 | 0 ± 0 | 0.03 ± 0.01 | 0.03 ± 0.02 | 0.01 ± 0.01 |
| **Lungs** | 0.3 ± 0.2 | 0.08 ± 0.06 | 0.03 ± 0.02 | 0.2 ± 0.03 | 0.13 ± 0.06 | 0.07 ± 0.02 |
| **Liver** | 0.8 ± 0.8 | 0.09 ± 0.01 | 0.06 ± 0.01 | 0.4 ± 0.2 | 0.5 ± 0.2 | 0.44 ± 0.07 |
| **Spleen** | 0.5 ± 0.3 | 0.08 ± 0.02 | 0.07 ± 0.02 | 0.25 ± 0.03 | 0.15 ± 0.04 | 0.11 ± 0.07 |
| **Pancreas** | 2 ± 1 | 0.1 ± 0.05 | 0.04 ± 0.02 | 0.2 ± 0.09 | 1.8 ± 0.9 | 0.33 ± 0.09 |
| **Stomach** | 0.5 ± 0.6 | 0.09 ± 0.07 | 0.02 ± 0.01 | 0.2 ± 0.01 | 1 ± 0.4 | 0.1 ± 0.1 |
| **Small Intestines** | 0.8 ± 0.6 | 0.06 ± 0.05 | 0.03 ± 0.02 | 0.2 ± 0.1 | 0.6 ± 0.1 | 0.08 ± 0.09 |
| **Kidneys** | 8 ± 1 | 3.3 ± 0.2 | 2.3 ± 0.4 | 4 ± 1.7 | 6 ± 1.6 | 3.6 ± 0.9 |
| **Tumour** | 1.1 ± 0.3 | 5 ± 1 | 1.5 ± 0.7 | 1.2 ± 0.6 | 20 ± 5 | 15 ± 4 |
| **Muscle** | 0.06 ± 0.06 | 0.03 ± 0.02 | 0.01 ± 0.01 | 0.07 ± 0.02 | 0.06 ± 0.08 | 0.02 ± 0.01 |
| **Bone** | 0.09 ± 0.07 | 0.04 ± 0.01 | 0.03 ± 0.03 | 0.25 ± 0.04 | 0.13 ± 0.08 | 0.07 ± 0.03 |
| **GI** | 0.5 ± 0.0 | 0.5 ± 0.2 | 0.1 ± 0.05 | 0.8 ± 0.7 | 0.8 ± 0.2 | 0.13 ± 0.03 |
| **Carcass** | 1 ± 0.0 | 0.6 ± 0.1 | 0.28 ± 0.04 | 0.6 ± 0.1 | 1.3 ± 0.8 | 0.54 ± 0.06 |

**Table S3.** Biodistribution profile of [^177^Lu]Lu-AU-SAR-M1 and [^177^Lu]Lu-AU-SAR-M2 in PC-3 xenograft bearing mice at 4 h and 23 h pi. All animals were pre-treated with Entresto as a means of in situ inhibition of NEP. Block groups received in addition a 100× molar excess of GRPR-blocking agent. Results are given as percentage of injected activity per gram of tissue (%IA/g) with the exception of carcass and gastrointestinal track (GI) which are given as percentage of injected activity (%IA).

|  | **[^177^Lu]Lu-AU-SAR-M1** | | | **[^177^Lu]Lu-AU-SAR-M2** | | |
| --- | --- | --- | --- | --- | --- | --- |
| **Organs** | **4 h Block** | **4 h** | **23 h** | **4 h Block** | **4 h** | **23 h** |
| **Blood** | 0.01 ± 0.00 | 0.03 ± 0.01 | 0.01 ± 0.00 | 0.01 ± 0.00 | 0.02 ± 0.01 | 0.01 ± 0.03 |
| **Lungs** | 0.04 ± 0.03 | 0.06 ± 0.03 | 0.06 ± 0.04 | 0.2 ± 0.2 | 0.08 ± 0.01 | 0.04 ± 0.03 |
| **Liver** | 0.08 ± 0.01 | 0.1 ± 0.01 | 0.07 ± 0.01 | 0.2 ± 0.02 | 0.2 ± 0.02 | 0.18 ± 0.01 |
| **Spleen** | 0.05 ± 0.02 | 0.07 ± 0.02 | 0.04 ± 0.03 | 0.08 ± 0.02 | 0.11 ± 0.03 | 0.09 ± 0.02 |
| **Pancreas** | 0.14 ± 0.02 | 3 ± 1.4 | 0.22 ± 0.07 | 0.17 ± 0.02 | 3 ± 1 | 0.35 ± 0.06 |
| **Stomach** | 0.13 ± 0.06 | 1.2 ± 0.3 | 0.14 ± 0.06 | 0.12 ± 0.04 | 1 ± 0.4 | 0.1 ± 0.04 |
| **Small Intestines** | 0.06 ± 0.03 | 0.9 ± 0.4 | 0.09 ± 0.08 | 0.07 ± 0.02 | 0.4 ± 0.1 | 0.07 ± 0.09 |
| **Kidneys** | 2.6 ± 0.3 | 2.83 ± 0.04 | 1.6 ± 0.2 | 4 ± 1 | 3.8 ± 0.6 | 3 ± 0.4 |
| **Tumour** | 0.7 ± 0.2 | 16 ± 4 | 8 ± 2 | 1.3 ± 0.6 | 15 ± 0.4 | 11 ± 1 |
| **Muscle** | 0.02 ± 0.02 | 0.02 ± 0.01 | 0.01 ± 0.01 | 0.01 ± 0.01 | 0.02 ± 0.01 | 0.02 ± 0.01 |
| **Bone** | 0.02 ± 0.02 | 0.07 ± 0.02 | 0.08 ± 0.05 | 0.04 ± 0.02 | 0.06 ± 0.03 | 0.04 ± 0.04 |
| **GI** | 1 ± 0.4 | 1.2 ± 0.6 | 0.3 ± 0.2 | 0.5 ± 0.5 | 1 ± 0.3 | 0.19 ± 0.08 |
| **Carcass** | 0.5 ± 0.02 | 0.55 ± 0.04 | 0.39 ± 0.05 | 0.49 ± 0.07 | 0.64 ± 0.02 | 0.39 ± 0.03 |

**Table S4**. Estimation of area-under-the-curve (AUC) for tumours and kidneys for [^177^Lu]Lu-AU-RM26-M2, [^177^Lu]Lu-AU-RM26-M4, [^177^Lu]Lu-AU-SAR-M1 and [^177^Lu]Lu-AU-SAR-M2 and their ratio, based on the data from the biodistributions and fitting of an one-phase decay elimination model.

| **AUC** | **AU-RM26-M2** | **AU-RM26-M4** | **AU-SAR-M1** | **AU-SAR-M2** |
| --- | --- | --- | --- | --- |
| **Tumour** | 62 | 331 | 242 | 259 |
| **Kidneys** | 56 | 97 | 45 | 68 |
| **Tumour/Kidneys** | 1.1 | 3.4 | 5.4 | 3.8 |
